# High hsa_circ_0020123 expression indicates poor progression to non-small cell lung cancer by regulating the miR-495/HOXC9 axis

**DOI:** 10.18632/aging.103722

**Published:** 2020-09-14

**Authors:** Rui Bi, Wei Wei, Yunhui Lu, Fengqing Hu, Xuhui Yang, Yuan Zhong, Lifei Meng, Mingsong Wang, Lianyong Jiang, Xiao Xie

**Affiliations:** 1 Department of Cardiothoracic Surgery, Xinhua Hospital, Shanghai Jiaotong University School of Medicine, Shanghai 200092, China; 2 Department of Cardiothoracic Surgery, Jinling Hospital, Nanjing University School of Medicine, Nanjing 210002, China; 3 Department of Nephrology, Changzheng Hospital, Shanghai 200003, China

**Keywords:** hsa_circ_0020123, miR-495, HOXC9, NSCLC, metastasis and proliferation

## Abstract

Circular RNAs (circRNAs) belong to non-protein-coding RNAs that regulate different pathophysiological procedures. Upregulation of hsa_circ_0020123 is found in non-small cell lung cancer (NSCLC); however, its activity and functions are not clear. In this study, the results showed that hsa_circ_0020123 expression increased in both tumor tissues and NSCLC cells. A higher hsa_circ_0020123 expression also led to poor prognoses among NSCLC patients assayed via FISH. The data of FISH also confirmed that hsa_circ_0020123 primarily had a cytoplasmic location. Hsa_circ_0020123 knockdown caused a significant decrease in nude mouse xenograft growth. Bioinformatics analyses and dual luciferase reporter assays confirmed that hsa_circ_0020123 was an miR-495 sponge and that the *HOXC9* gene was a miR-495 target. The miR-495 downregulation reversed cell migration and proliferation inhibition induced by hsa_circ_0020123 silencing *in vitro*. HOXC9 overexpression reversed miR-495-induced inhibition of cell migration and proliferation. The dual luciferase reporter assay demonstrated that hsa_circ_0020123 interacted with miR-495 by binding to the HOXC9 3′-UTR to suppresses post-transcriptional HOXC9 expression. Taken together, our study found that hsa_circ_0020123 functioned like a tumor promoter via a novel hsa_circ_0020123/miR-495/HOXC9 axis, highlighting its possibility as a new NSCLC therapeutic target.

## INTRODUCTION

Non-small cell lung cancer (NSCLC) includes around 80% of lung cancers, which is a main factor regarding cancer-associated mortality in China [1, 2]. Despite treatment advances, overall NSCLC 5-year survival remains low [3]. PET-CT imaging and pathological examination are usually used as diagnostic criteria for NSCLC. Accuracy and speed are severely constrained. Thus, the identification of diagnostic markers that improve early detection will expand the opportunities for surgical intervention in NSCLC patients.

Circular RNA (circRNA) is an endogenous RNA that consists of a covalently closed continuous loop. The activity of circRNAs has been reported in the tumorigenesis of hepatocellular carcinoma [4], oral squamous cell carcinoma [5], gastric cancer [6], glioma [7], as well as other human cancers [8–10]. For example, circRNA SMARCA5 inhibits migration, proliferation, and invasion of NSCLC through miR-19b-3p/HOXA9 axis [11]. CircRNA PRMT5 facilitates NSCLC proliferation via upregulating EZH2 through sponging miR-377/382/498 [11]. Upregulation of circ_0016760 has an unfavorable prognosis among NSCLC patients, which enhances cancer progression via the miR-1287/GAGE1 axis [12]. Hsa_circ_0020123 exerts oncogenic properties through suppression of miR-144 in NSCLC [13]. At least two human circRNAs, hsa_circ_0007385 and hsa_circ_0020123, have been identified in microarrays as oncogenes promoting NSCLC tumorigenesis [13, 14]. Currently, it is hypothesized that competing endogenous RNAs (ceRNAs), e.g. lncRNAs, mRNAs, and pseudogenes, might combine together and modulate other components through competitively binding to microRNA response elements (MREs) [15]. Studies report that circRNAs could serve as ceRNAs, which would prevent miRNAs from accessing the target genes.

The current study found that hsa_circ_0020123 had abnormal expression in NSCLC in patients. However, the function and regulatory mechanism of hsa_circ_0020123 as a ceRNA in NSCLC progression are still unclear. Therefore, we examined the biological function of hsa_circ_0020123 and it underlying molecular mechanisms in NSCLC development from multiple viewpoints.

## RESULTS

### Hsa_circ_0020123 downregulation suppresses tumor growth in nude mouse xenografts

**Figure 1:**
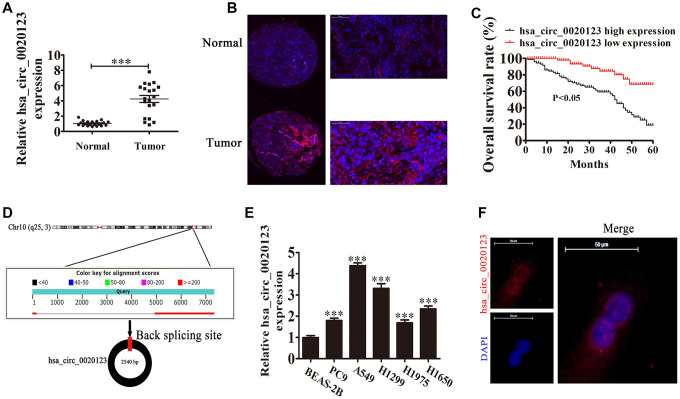
**hsa_circ_0020123 expression is increased in non-small cell lung cancer (NSCLC) tissues and cell lines.** (**A**) qRT-PCR assay of hsa_circ_0020123 in 20 paired NSCLC tumor tissues and adjacent non-tumor tissues. Data are the means ± SD. ^***^P < 0.001 vs. normal controls. (**B**) Fluorescent *in situ* hybridization (FISH) indicates the subcellular localization of hsa_circ_0020123. (**C**) The prognostic significance of hsa_circ_0020123 expression for hsa_circ_0020123 patients was performed with FISH values using the median value as the cut-off. The observation time was 60 months. (**D**) Genomic loci of *HOXC9* and hsa_circ_0020123. The red signal indicates back splicing. (**E**) qRT-PCR assay of hsa_circ_0020123 in A549, PC9, H1299, H1975, H1650, and BEAS-2B normal lung epithelial cells. Data are means ± SD. ***P < 0.001 vs. normal controls. (**F**) Fluorescent *in situ* hybridization indicates the subcellular localization of hsa_circ_0020123.

Previous microarray profiles revealed that hsa_circ_0020123 was upregulated in NSCLC tissues [14]. Our present research results also found that hsa_circ_0020123 expression increased in NSCLC tumor tissues compared with adjacent normal tissues using both qPCR and FISH detection assays (*P*<0.001) (Figure 1A and 1B). FISH detection also showed that hsa_circ_0020123 was predominantly located in the cytoplasm. We divided samples into relatively high (above the adjacent normal tissue levels; n = 38) and relatively low (below the adjacent normal tissue levels; n = 42) expression levels. There was no correlation between hsa_circ_0020123 expression and clinical factors, such as sex (males and females) and patient age (≤ 60 years and > 60 years). However, the high hsa_circ_0020123 expression was positively correlated to lymph node invasiveness, TNM stage, and tumor size (Table 1). Also, the Gehan-Breslow-Wilcoxon Test survival curves validated that NSCLC patients with high hsa_circ_0020123 expression in NSCLC exhibited low overall survival (*P*<0.05) (Figure 1C). The results suggested that hsa_circ_0020123 expression had an indispensable function in NSCLC progression.

**Table 1:** The clinic-pathological factors of 80 NSCLC patients.

**Characteristics**	**Numbers**	**Expression of circRNA ARHGAP10**		**P value**
		**Low (N = 42)**	**High (N = 38)**	
**Sex**				**0.238**
male	48	26	22	
female	32	16	16	
**Age**				**0.314**
≤60	39	24	15	
>60	41	18	23	
**TNM stage**				**0.016**
I and II	43	26	17	
III and IV	37	16	21	
**Lymph node metastasis**				**0.038**
negative	46	30	16	
positive	34	12	22	
**Tumor size**				**0.034**
≤ 3 cm	48	32	16	
> 3 cm	32	10	22	

We also found that hsa_circ_0020123 was derived and cyclized from a portion of the *PDZD8* gene exon and was located at chr10:119042605-119049859 (Figure 1D). qRT-PCR confirmed that hsa_circ_0020123 expression increased in NSCLC cell lines (H1299, A549, H1975, PC9, and H1650), compared with normal human lung epithelial cells (BEAS-2B) (*P*<0.001) (Figure 1E). The highest expression was in H1299 and A549 cells, which were used in the subsequent assays. FISH showed that hsa_circ_0020123 was predominantly located in cytoplasm (Figure 1F).

**Figure 2:**
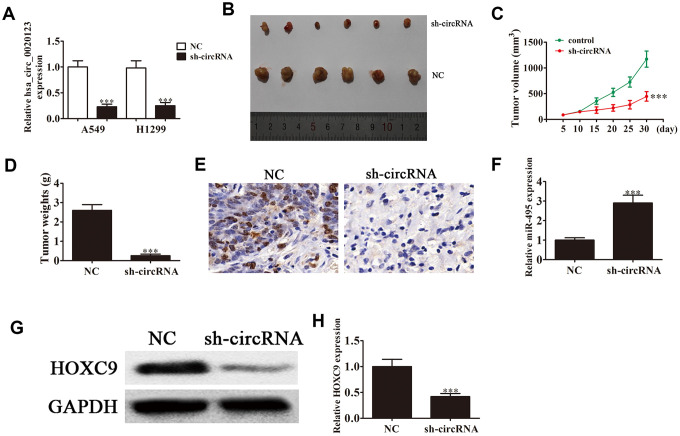
**Downregulation of hsa_circ_0020123 suppresses tumor formation in nude mouse xenografts.** (**A**) qRT-PCR shows expression of hsa_circ_0020123 in adenovirus-transfected (sh-hsa_circ_0020123) and circRNA control-transfected (sh-NC) A549 and H1299 cell lines. (**B**) Representative images of nude mouse xenografts of A549 cells (n = 6). (**C**) Tumor volumes in mice were measured every 5 days. Data are means ± SD. ***P < 0.001 vs. controls. (**D**) Tumor weight was measured 30 days after grafting. Data are the means ± SD. ***P < 0.001 vs. controls. (**E**) Immunohistochemical staining of Ki67 in tumor tissues from both sh-NC and sh-circRNA groups.(**F**) qRT-PCR assay of miR-495 expression. Data are means ± SD. ***P < 0.001 vs. controls. (**G**, **H**) Western blot assay of HOXC9 protein expression in tumor tissues. Data are means ± SD. ***P < 0.001 vs. controls.

Lentivirus-mediated silencing decreased hsa_circ_0020123 expression (*P*<0.001) (Figure 2A), and we used A549 cells transfected with hsa_circ_0020123 lentiviral interference vectors to assay tumor formation in nude mouse xenografts. Tumor volumes were measured 5 days after grafting with a vernier caliper. hsa_circ_0020123 knockdown clearly resulted in reduced xenograft volume and weight (*P*<0.001) (Figure 2B–2D). Immunohisto-chemical staining revealed a decrease in the Ki67-positivity, consistent with suppression of tumor growth by downregulation of hsa_circ_0020123 (Figure 2E). qRT-PCR found that miR-495 expression increased in tumor tissues with hsa_circ_0020123 silencing (*P*<0.001) (Figure 2F). Knockdown of hsa_circ_0020123 inhibited HOXC9 protein expression as shown in western blots (*P*<0.001) (Figure 2G and 2H), consistent with a NSCLC tumorigenesis role.

### hsa_circ_0020123 knockdown suppresses NSCLC cell invasion and proliferation via miR-495 sponge activity

**Figure 3:**
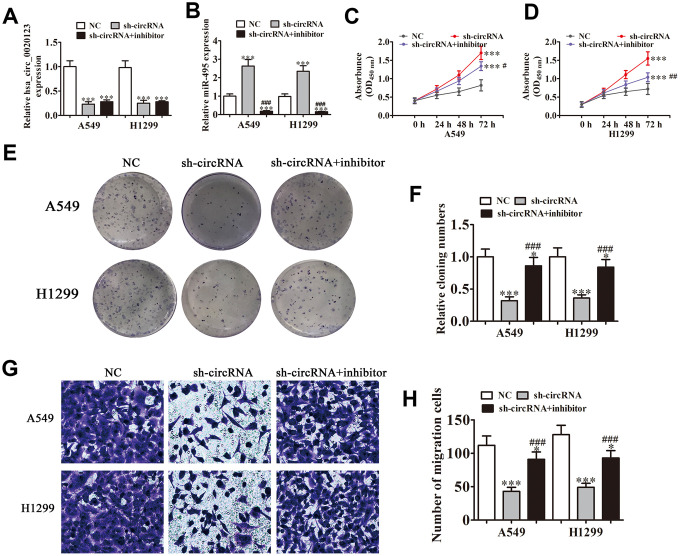
**Knockdown of hsa_circ_0020123 suppresses proliferation and invasiveness of NSCLC cells induced by miR-495 sponging.** (**A**, **B**) qRT-PCR assay of hsa_circ_0020123 expression (**A**) and miR-495 expression (**B**) after transfection with sh-hsa_circ_0020123 and miR-495 inhibitor, either singly or in combination. Data are means ± SD. ***P < 0.001 vs. controls. (**C**, **D**) CCK8 assay of cell proliferation. Data are means ± SD. ***P < 0.001 vs. controls. ^#^P < 0.05, ^##^P < 0.01 vs. sh-circRNA. (**E**, **F**) Colony formation assay of A549 and H1299 cell proliferation. Data are means ± SD. *P < 0.05, ***P < 0.001 vs. controls. ^###^*P* < 0.001 vs. sh-circRNA. (**G**, **H**) Transwell assay of A549 cell migration. Data are means ± SD. *P < 0.05, ***P < 0.001 vs. controls. ^###^*P* < 0.001 vs. sh-circRNA.

Statistical analysis showed that miR-495 was the downstream target of hsa_circ_0020123. qRT-PCR verified that hsa_circ_0020123 expression was suppressed significantly by lentivirus-mediated silencing. miR-495 suppression had no effects on hsa_circ_0020123 expression (Figure 3A), and hsa_circ_0020123 downregulation significantly promoted miR-495 expression. The miR-495 inhibitor significantly reduced miR-495 expression (Figure 3B). The CCK8 and colony formation assays verified that hsa_circ_0020123 silencing suppressed A549 and H1299 cell proliferation and miR-495 inhibition reversed hsa_circ_0020123 silencing-induced inhibition of proliferation (Figure 3C–3F). The A549 and H1299 cell migration was inhibited by hsa_circ_0020123 silencing but restored by decreased miR-495 expression (Figure 3G, 3H). The overall results showed that hsa_circ_0020123 silencing inhibited NSCLC cell migration and proliferation via miR-495 promotion, but the mechanism remains to be established.

### HOXC9 overexpression reverses miR-495-induced inhibition of NSCLC cell migration and growth *in vitro.*

**Figure 4:**
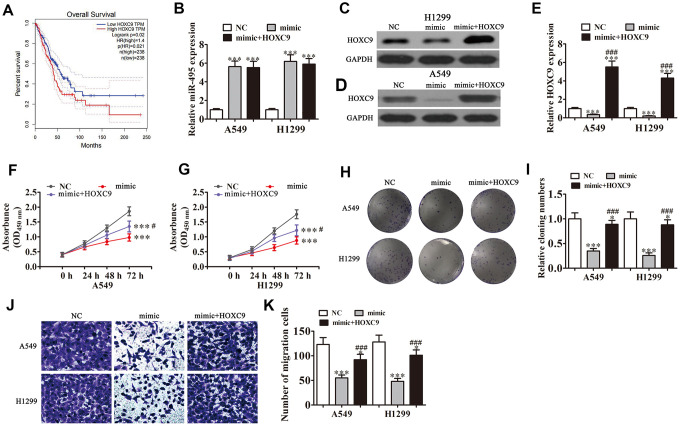
**Overexpression of HOXC9 reverses miR-495-induced inhibition of NSCLC cell growth and migration *in vitro*.** A549 and H1299 cells were transfected with miR-495 mimics or a HOXC9 overexpression vector either singly or in combination. (**A**) GEPIA analysis of survival of NSCLC patients with high versus low HOXC9 expressing cases. (**B**) qRT-PCR assay of miR-495 expression in A549 and H1299 cells. Data are means ± SD. ***P < 0.001 vs. controls. (**C**, **D**) Western blots of HOXC9 protein expression in A549 (**C**) and H1299 (**D**) cells. (**E**) Relative protein expression is reported as means ± SD. ***P < 0.001 vs. controls; ^###^*P* < 0.001 vs. mimic. (**F**, **G**) CCK8 assay of A549 (**F**) and H1299 (**G**) cell proliferation. Data are means ± SD. ***P < 0.001; ^#^*P* < 0.05 vs. mimic.(**H**, **I**) Colony formation assays of A549 and H1299 cells proliferation. Data are means ± SD. *P < 0.05, ***P < 0.001 vs. controls;^###^*P* < 0.001 vs. mimic. (**J**, **K**) Transwell assays of A549 and H1299 cell migration and invasiveness. Data are means ± SD. *P < 0.05,***P < 0.001 vs. controls; ^###^*P* < 0.001 vs. mimic.

Bioinformatics analyses indicated that high HOXC9 expression correlated to a poor prognosis in NSCLC patients (*P*<0.001) (Figure 4A). qRT-PCR found that transfection with miR-495 mimics promoted miR-495 expression in both H1299 and A549 cells (*P*<0.001). HOXC9 overexpression had no effect on miR-495 level after overexpression of miR-495 (*P*<0.001) (Figure 4B), indicating that HOXC9 was a downstream miR-495 target. Western blot assays verified that miR-495 overexpression inhibited HOXC9 protein expression, but after transfection with HOXC9, overexpression of vector promoted HOXC9 expression even after miR-495 overexpression (Figure 4C–4E). Colony formation assays and CCK8 assays found that HOXC9 overexpression reversed miR-495-induced inhibition of A549 and H1299 cell proliferation (Figure 4F–4I). H1299 and A549 cell Transwell migration was inhibited in cells that overexpressed miR-495 but was restored in cells that overexpressed HOXC9 (Figure 4J, 4K), consistent with miR-495 targeting of HOXC9.

### Interactions of hsa_circ_0020123, miR-495, and HOXC9

**Figure 5:**
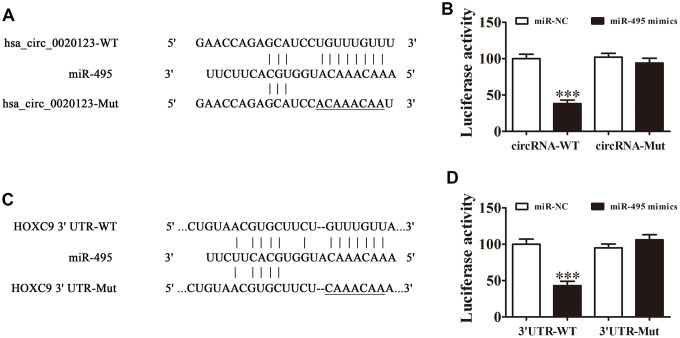
**Interactions among hsa_circ_0020123, miR-495 and HOXC9.** (**A**) Prediction of binding sites of miR-495 in hsa_circ_0020123. The mutant (MUT) version of hsa_circ_0020123 is presented. (**B**) Relative luciferase activity determined 48 h after transfection of HEK293T cells with miR-495 mimic/NC or hsa_circ_0020123 wild-type (WT)/Mut. Data are presented as means ± SD. ***P < 0.001. (**C**) Prediction of binding sites of miR-495 within the 3'UTR of HOXC9. The MUT version of 3'-UTR-HOXC9 is shown. (**D**) Relative luciferase activity determined 48 h after transfection of HEK293T cells with miR-495 mimic/NC or 3'UTR-HOXC9 WT/Mut. Data are presented as means ± SD. ***P < 0.001.

Bioinformatics analysis of the interactions of the miR-495, hsa_circ_0020123, and HOXC9 indicated that hsa_circ_0020123 targeted miR-495. The results from luciferase reporter assays indicated that miR-495 was a downstream hsa_circ_0020123 binding target (site: 986-1009 of hsa_circ_0020123) (Figure 5A) and hsa_circ_0020123 inhibited luciferase activity in WT but not in mutated cell lines (Figure 5B). Bioinformatics analysis also indicated that HOXC9 was a miR-495 target, showing that miR-495 interacted directly with HOXC9 3'-UTR (site: 594-616 of HOXC9 3'-UTR) to suppress expression of its mRNA (Figure 5C). In the luciferase reporter assay, miR-495 inhibited luciferase activity in WT but not mutant (MUT) cell lines (Figure 5D). Combined results indicated that hsa_circ_0020123 silencing inhibited NSCLC invasiveness and growth with targeting of the miR-495/HOXC9 axis.

## DISCUSSION

CircRNAs have gained attention due to their regulation and functional roles in human cancers. The hsa_circ_0020123 expression increased in both NSCLC cell lines and tissues. High hsa_circ_0020123 expression correlated with a poor prognosis. In this investigation we suggested that hsa_circ_0020123 expression increased in NSCLC tissues. Higher expression predicted a higher grade, higher aggression (lymph node invasiveness), and tumor size, suggesting that hsa_circ_0020123 functioned in NSCLC regulation of invasiveness and proliferation. Further investigations found that hsa_circ_0020123 silencing suppressed tumor growth in nude mouse xenografts, suggestive of a role in tumorigenesis; it also promoted miR-495 expression *in vitro*. The miR-495 inhibition reversed the cell proliferation and migration inhibition that resulted from hsa_circ_0020123 silencing. The results supported the hypothesis that hsa_circ_0020123 played a role as a molecular sponge to suppress the biological activity of miR-495; a relationship that was further validated in a dual luciferase reporter assay.

MiRNAs are expressed abnormally in a number of human cancers and they function in tumorigenesis and tumor development [19–21]. Downregulation of miR-495 has previously been reported in melanoma cell lines and tissues, and miR-495 overexpression has been associated with suppression of cell proliferation and invasiveness of melanoma cells [22]. miR-495 was found to suppress hepatocellular carcinoma cell invasion and proliferation through targeting directly insulin-like growth factor receptor-1 [23]. Previous studies have found that miR-495 targets MTA3 in the regulation of lung cancer growth and migration [24]. miRNAs are post transcriptional inhibitors of gene expression, binding to the mRNAs 3′-UTRs. In this study, miR-495 suppressed HOXC9 expression, and HOXC9 overexpression reversed miR-495-induced inhibition of cell migration and proliferation, validating that HOXC9 was a miR-495 target, which was confirmed with the dual luciferase reporter assay.

*HOX*, or homeobox, genes act as transcription factors. In humans, 39 *HOX* genes have been identified and organized into 13 paralogous groups and four clusters, including HOXB, HOXC, HOXA, and HOXD. HOXC9 is upregulated in breast cancer, whose downregulation suppresses tumor cell proliferation and invasiveness [25, 26]. The present results are consistent with former findings that HOXC9 promotes the invasiveness and proliferation of NSCLC cells. miR-495 bound to the HOXC9 3'-UTR region to suppress its expression, suggesting a role as a tumor suppressor in the miR-495/HOXC9 pathway in NSCLC.

## CONCLUSIONS

Hsa_circ_0020123 promoted oncogenesis via functioning as a miR-495 sponge, and may be a newly identified prognostic marker in NSCLC. Targeting the novel hsa_circ_0020123/miR-495/HOXC9 axis represents a potential therapeutic strategy for NSCLC.

## MATERIALS AND METHODS

### Tissue samples

In summary, we collected 92 fresh NSCLC tissues as well as paired adjacent noncancerous lung tissues after obtaining informed patient consent at the Xinhua Hospital, Shanghai Jiaotong University School of Medicine, Shanghai, China. We evaluated pathological and histological diagnostics of NSCLC patients following the Revised International System for Staging Lung Cancer (RISSL). Patients did not receive any chemotherapy or radiotherapy before tissue sampling (Table 1). We snap-froze samples in liquid nitrogen and stored them at -80°C prior to RNA extraction. The Ethics Committee in Xinhua Hospital, Shanghai Jiaotong University School of Medicine approved the investigation.

### Experimental procedures

The Ethics Committee in Xinhua Hospital, Shanghai Jiaotong University School of Medicine approved all experiments performed in animals. We purchased 12 BALB/c nude mice, 4 weeks of age, weighing 15~20 g from SLARC, Shanghai. We purchased lung cancer cell lines H1975, H1299, PC9, A549, and H1650, and the normal lung epithelial cell line, BEAS-2B from the American Type Culture Collection (Manassas, VA, USA). We cultured cells in Dulbecco’s Modified Eagle’s Medium (DMEM; Gibco, Gaithersburg, MD, USA) with fetal bovine serum (FBS; Gibco, Gaithersburg, USA), 100 IU/mL of 10%, penicillin, and 100 μg/mL streptomycin at 37°C and 5% CO_2_. We transfected small interfering RNAs (siRNAs), miR-495 inhibitors, miR-495 mimics, a HOXC9 overexpression vector, and the negative controls into cultured A549 or H1299 cells by Lipofectamine 2000 (Invitrogen, Carlsbad, USA) following the standard kit procedures. To investigate hsa_circ_0020123 activity *in vivo*, stably transfected hsa_circ_0020123-silenced A549 cells were generated. Hsa_circ_0020123-targeting shRNA, miR-495 mimic, miR-495 inhibitor was synthesized by Sengong Biotech (Shanghai, China), with the following sense sequence: sh-circRNA: forward: 5'-GUCCAAAGAUGGAAAUAAAUU-3', reverse: 5'-AAAUAAAGGUAGAAACCUGUU-3'. miR-495 mimic: 5'-AAACAAACAUGGUGCACUUCUU-3'. miR-495 inhibitor: 5'-AGGCCGCCACCCGCCCGCGAUCCCU-3'. We purchased overexpression plasmid of HOXC9 (pcDNA3.1-HOXC9) and hsa_circ_0020123 (pcDNA3.1-hsa_circ_0020123), and empty plasmid (pcDNA3.1) from GeneChem (Shanghai, China).

### Bioinformatics analysis

We performed analysis and hsa_circ_0020123-miRNA-target gene activity predictions with the *Circular RNA Interactome*. Prediction of the miR-495 mRNA target gene was conducted with *TargetScanHuman*. Expression of *HOXC9* gene expression and patient prognosis were estimated using GEPIA.

### Clone formation assays and cell proliferation

We assayed cell proliferation with a Cell Counting Kit-8 (CCK-8; Invitrogen, Carlsbad, CA, USA). We seeded transfected cells into plates with 96-wells in triplicate at 2,000 cells per well and assayed viability at 0, 24, 48, 72, and 96 h after seeding them, following the standard procedures of the kit. Colony formation was assayed in transfected cells seeded into plates with 6 wells at 2,000 cells/well and grown in DMEM with 10% FBS for 10 days. We counted and photographed colonies after fixing and staining.

### Fluorescence *in situ* hybridization (FISH)

We utilized hsa_circ_0020123-specific FITC-labeled probes for *in situ* hybridization [16]. We counterstained nuclei with 4,6-diamidino-2-phenylindole (DAPI). We performed the procedures following the kit manufacturer’s instructions (Genepharma, Shanghai, China).

### Quantitative reverse transcription-polymerase chain reaction (qRT-PCR)

We extracted RNA with TRIzol reagent (Invitrogen, Carlsbad, CA, USA) and synthesized cDNA with a pTRUEscript First Strand cDNA Synthesis Kit (Aidlab, Beijing, China). We performed qRT-PCR with 2× SYBR Green qPCR Mix (Invitrogen, Carlsbad, CA, USA) with an ABI 7900HT qPCR system (Thermo Fisher Scientific, Waltham, USA). We determined fold-change in expression via the 2^−ΔΔCT^ method. qRT-PCR amplification was performed using primers: hsa_circ_0020123: forward: 5′-CTTCTCCAGTTACTTGCTTGTGTAAG-3′; reverse: 5′-GTATCTACTGTCAACCCGGCAG-3′; miR-495: forward: 5′-ACACTCCAGCTGGGAAACAAACATGGTGCA-3′; reverse: 5′-CTCAACTGGTGTCGTGGAGTCGGCAATTCAGTTGAGAAGAAGTG-3′; U6: forward: 5′-CTCGCTTCGGCAGCACA-3′; reverse: 5′-AACGCTTCATTTGCGT-3′; GAPDH: forward: 5′-AATCCCATCACCATCTTCC-3′; reverse: 5′-CATCACGCCACAGTTTCC-3′. FOSL2 and hsa_circ_0020123 expression levels were normalized to GAPDH and miR-495 expression level was normalized to U6.

### Cell migration assay

We assayed cell migration in Transwell chambers with 24 wells with 8 μm pore-size membranes (BD Biosciences, Franklin Lakes, USA). In brief, we added 1 × 10^5^ cells in 200 μL serum-free medium to the upper chamber. We filled lower chamber with 500 μL complete medium as a chemoattractant. We fixed the cells that had invaded the lower chamber after 1 d with 4% paraformaldehyde for 0.5 h and stained them with Crystal Violet for 10 minutes.

### Invasiveness assay and tumor xenograft formation

We injected a total of 1 × 10^7^ viable wild-type (WT) or siRNA A549 cells into nude mice right flanks [17]. We measured tumor sizes every 5 days with a vernier caliper and calculated the volume as V = 0.5 × length × width^2^. Mice were euthanized and samples prepared for qRT-PCR and Ki67 immunohistochemical staining 30 days after implantation.

For invasiveness analysis, WT and siRNA A549 cells (2×10^5^) were transfected with luciferase expression vectors, and the cells were injected intravenously into the tails of mice. After 30 d, A549 cell invasiveness was analyzed by bioluminescence imaging following an intravenous injection of luciferin (150 mg luciferin/kg body weight) into the tails.

### Western blot assays

We extracted protein from cells or tissues with RIPA lysis buffer. Western blot assays were performed following a previous procedure [18]. We separated protein samples (20 μg) using a 10% sodium dodecyl sulfate polyacrylamide electrophoresis (SDS-PAGE) gel and transferred the proteins to a polyvinylidene fluoride (PVDF) membrane (Bio-Rad Laboratories, Hercules, CA). We blocked membranes with nonfat dry milk (Yili Milk Company, Inner Mongolia, China) at room temperature for 2 hours, and then hybridized with anti-HOXC9 (1:1000 dilution; SC-81100; Santa Cruz Biotechnology, CA.USA) and anti-GAPDH antibodies (1:2000 dilution; SC-365062; Santa Cruz Biotechnology) at 4°C overnight. We next added secondary biotin-conjugated antibodies, and developed immunoreactive bands with the enhanced chemiluminescence kit (Thermo Fisher Scientific). We used GAPDH as a loading control.

### Dual luciferase reporter assay

We produced reporter plasmids through inserting circRNA or the HOXC9 3'-UTR sequence into a pmirGLO vector (Promega, Madison, USA). We cotransfected reporter plasmids and miR-495 mimics into human embryonic kidney (HEK) 239T cells through using Lipofectamine 2000. After cultivation for 2 d, we determined firefly and Renilla luciferase activities with a Dual Luciferase Reporter Assay System (Promega, Sunnyvale, USA) following standard protocols.

### Statistical analyses

We reported data as the means ± standard deviation (SD). We used GraphPad Prism version 5.0 (GraphPad, La Jolla, USA) to compare differences between groups. *P*-values ≤ 0.05 were considered as statistical significance.

### Ethics approval

The Animal Research Committee of Xinhua Hospital, Shanghai Jiaotong University School of Medicine (Shanghai, China) approved all experimental protocols and surgical procedures.
